# Meeting report from the Companion Animal Genetic Health conference 2018 (CAGH 2018): a healthy companionship: the genetics of health in dogs

**DOI:** 10.1186/s40575-018-0061-0

**Published:** 2018-08-29

**Authors:** Sally L. Ricketts, Thomas W. Marchant

**Affiliations:** 1 Canine Genetics Research Group, Kennel Club Genetics Centre, Animal Health Trust, Kentford, Newmarket, Suffolk, UK; 2 The Royal (Dick) School for Veterinary Studies and Roslin Institute, University of Edinburgh, Easter Bush, Midlothian, UK

## Plain English summary

The first meeting for the Companion Animal Genetic Health (CAGH) conference invited scientists from around Europe interested in dog and cat health to discuss their research. The meeting was open to all aspects of companion animal research but discussions mainly covered three central themes across the conference. The first was the importance of obtaining high-quality data for the characteristics that are under investigation. Examples presented were appetite, cardiac measurement, and skull shape, which along with genetic data can be used to investigate specific disorders. Detailed owner questionnaires formed a large part of these endeavours and are crucial to generate detailed information about certain characteristics such as behaviour. The second theme centred on the use of whole genome (DNA) sequences to investigate a range of inherited diseases and the importance of the field’s successful international data sharing for this purpose. Talks given also highlighted the need for an updated reference dog DNA sequence and tools to help identify more complex DNA variation (i.e. not simple single-base (letter) changes; larger insertions or deletions of DNA of more than 1000 bases for example). Lastly, there is now a real focus on understanding the biological processes underlying a mutation that has been associated with disease. As well as informing veterinary and possibly also human medicine, these studies will be important for complex diseases such as cancer that may not be amenable to DNA tests, but that research will inform in terms of diagnosis and treatment.

## Introduction

This inaugural meeting of geneticists, clinicians and epidemiologists encompassed discussion, presentations and abstracts from researchers working predominantly in the field of canine genetics and health, but also included representatives working in feline genomics. The conference sought to bring together researchers in the field from around Europe to share their latest research findings, discuss new techniques and establish new collaborations. Dr. Cathryn Mellersh and Dr. Jeffrey Schoenebeck hosted the two-day meeting which saw forty-five delegates from seven European countries present seventeen short talks and nine posters largely covering canine research but also including feline abstracts. Dr. Robert Ogden, senior lecturer and Head of Conservation Genetics at the University of Edinburgh, gave an intriguing keynote lecture on his work using animal genetics in molecular ecology, population genetics and conservation projects to guide species management and law enforcement across the world. The Royal (Dick) School for Veterinary Studies, the University of Edinburgh and Edinburgh Genomics helped sponsor the meeting. Following the success of this inaugural meeting, CAGH organisers are looking to repeat the event in the summer of 2020 with the ambition to appeal to the wider companion animal veterinary research community. We present here a summary of the meeting in which we will discuss its central themes, referring to the accompanying abstracts in this issue of the Journal, and the directions in which the field is moving in relation to these themes.

## Precision phenotype acquisition

CAGH enjoyed a diverse range of talks covering a wide variety of different traits, each of which requiring a unique and interesting method for phenotype capture. Precision phenotyping included using computer tomography (CT) scans to generate high resolution replicas of the canine skull to assist in fine-mapping the brachycephalic canine head shape locus. A complementary study then scored a variety of phenotypes of the upper airway to determine the genetic contributions to upper airway syndromes in brachycephalic and non-brachycephalic breeds (Marchant et al. O5). Several abstracts described the use of owner-targeted questionnaires to capture extensive datasets for canine traits and disease epidemiology in dogs. In addition, developments in using automated algorithms to ‘clean’ error-prone large survey datasets presented exciting possibilities for future survey-based studies to enrich and increase the power of their data collection (Woolley et al. P6).

## The use of whole genome sequencing to identify Mendelian disease variants

As anticipated and in line with human disease research, there is an increasing number of Mendelian (predominantly autosomal recessive) canine diseases for which the genetic cause is being characterised by whole genome sequencing (WGS) alone in the absence of any prior knowledge or mapping experiments. The WGS studies presented at this meeting sequenced one or more affected cases from a particular dog breed, and in two instances their first-degree unaffected relatives. Using in-house or collaborative WGS databases of animals from multiple breeds/cross breeds/canids, benign variants have been ‘filtered’ to focus in on a small number (< 20) of gene polymorphisms only found in cases. These studies have underlined the importance of the availability of collaborative and shared canine WGS databases, particularly the Dog Biomedical Variant Database Consortium (DBVDC) that was utilised in much of the research presented. The DBVDC is an international collaboration organised and curated by Professor Tosso Leeb and Dr. Vidhya Jagannathan at the University of Bern, Switzerland, that currently includes data from > 20 collaborators and is currently comprised of WGS data from over 550 purebred dogs and multiple cross breeds and wolves. Another growing WGS database that will also contribute to the DBVDC is the ‘Give a Dog a Genome’ project (Burmeister et al. P3). This project is led by Dr. Cathryn Mellersh and Dr. Louise Burmeister at the Kennel Club Genetics Centre at the Animal Health Trust, UK, and currently comprises WGS of one or more individuals of > 100 breeds. The extensive Cani-DNA biobank run by Dr. Catherine André at the University of Rennes, France has also contributed to research endeavours investigating both simple and complex canine genetic diseases (Botherel et al. P2; Correard et al. O9; Hédan et al. O3; Rimbault et al. O1). Excitingly, the results of these studies are now being translated to their human disease counterparts, with the potential for future impact on both the diagnosis and treatment of these conditions in both dogs and humans. This highlights a continuing important role of the pedigree dog in informing human disease and medicine.

Two studies presented at this meeting have found variants in novel genes not previously associated with the disease under investigation (Hitti, Oliver et al. O10; Jenkins et al. O6). Whilst the confirmation of these gene variants as mutations is somewhat dependent on both their disease co-segregation in extended breed-matched sample sets and the availability of relevant tissues for functional analyses to mitigate against false-positive/incidental findings, all of the above studies illustrate the power of using these multi-breed WGS databases to refine the number of variants that require ‘wet lab’ follow-up. It is very likely that the increased pace and cost-effectiveness of mutation identification of autosomal recessive canine diseases over the next few years will facilitate a move towards the development of a clinical sequencing initiative for dogs, with the necessary input of the referring specialist clinician for disease characterisation and to aid in the downstream clarification of genetic findings to clinical disease. With these cross-discipline collaborations, this should have a significant impact on mitigating the spread of new autosomal recessive mutations in the canine population, by WGS of the first emerging affected dogs.

## The requirement for an updated canine reference genome

There is a growing need for an updated canine reference genome, and this was highlighted by two talks in particular (Jenkins et al. O6; Marchant et al. O5) where gaps (bases denoted as N) in the current Boxer genome sequence were in areas of interest to the specific diseases studied. Mutations within these regions or in areas of incorrectly unannotated genome would not be picked up by the current variant filtering analysis pipelines that utilise predictive tools to prioritise variants. There are several initiatives underway internationally to resolve issues of gaps and annotation, including long-read sequencing of genomes and functional annotation using RNA sequencing data. Pooling of the data from these efforts alongside incorporation of published corrections of the reference sequence should enable a new release of the canine genome within the near future.

## Developing an automated analysis pipeline for structural variant discovery in canine WGS

Two talks in particular emphasised the need for automated data analysis pipelines to discover structural variation in canine whole genome sequences, as these are not currently being identified by current variant calling pipelines that are only able to detect short insertion-deletions of DNA sequence (Hédan et al. O3; Hitti, Oliver et al. O10). For efficient integration of collaborative datasets, harmonisation of approaches may be necessary to enable the effective variant filtering seen in the current single nucleotide polymorphism (SNP) calling datasets discussed above.

## Bridging genetics and function

Genome-wide association studies (GWAS) using high density SNP arrays continue to be common practice for investigating the underlying genetic associations with canine disease and morphological traits. Previously within the field, GWAS studies tended to report lists of associated variants due to practical experimental challenges. While this “GWAS tag and bag” culture has given insights into genomic regions of interest, it is easy to lose sight that an association is not necessarily equivalent to disease causality. A noticeable trend seen across the CAGH abstracts was an increased effort to bridge the gap between genomic loci/putatively causal variants and the effects they may have in a biological setting to establish functional causality. Cross-species sequence comparisons and simple *in-silico* techniques such as Sorting Intolerant from Tolerant (SIFT) and PolyPhen-2 begin to predict potentially damaging effects of variants (Correard et al. O9; Hitti, Oliver et al. O10; Jenkins et al. O6; Marchant et al. O5). Transcriptomic analyses have increased in popularity with multiple examples identifying splice variants or altered gene expression profiles between experimental cohorts (Jenkins et al. O6; Marchant et al. O5; Markby et al. O7; Rimbault et al. O1). Furthermore, several studies have also used mRNA sequencing to highlight errors in the current reference genome annotation, further urging the need for additional reference genomes (as discussed earlier). Interest was also expressed into using serum metabolomics and cell reporter assays to resolve the effects of variants on gene function and to broaden the toolbox of commonly used techniques within the field (Hitti, Burmeister et al. O8).

In summary, this meeting has demonstrated the high calibre of multi-disciplinary research being undertaken currently in the field of canine health and disease, with methodologies and approaches in line with equivalent human fields of research. As we now are seeing technologies such as WGS bringing much to the field of Mendelian disease genetics, it is envisaged that the field of complex disease research will in time follow, with effective international collaboration of research efforts.

